# Shape memory polymers with high and low temperature resistant properties

**DOI:** 10.1038/srep14137

**Published:** 2015-09-18

**Authors:** Xinli Xiao, Deyan Kong, Xueying Qiu, Wenbo Zhang, Yanju Liu, Shen Zhang, Fenghua Zhang, Yang Hu, Jinsong Leng

**Affiliations:** 1 Harbin Institute of Technology, Department of Chemistry, No. 92 West Dazhi Street, Harbin 150001, People’s Republic of China; 2 Harbin Institute of Technology, Centre for Composite Materials and Structures, No. 2 YiKuang Street, Harbin 150080, People’s Republic of China; 3 Harbin Institute of Technology, Department of Astronautical Science and Mechanics, No. 92 West Dazhi Street, Harbin 150001, People’s Republic of China

## Abstract

High temperature shape memory polymers that can withstand the harsh temperatures for durable applications are synthesized, and the aromatic polyimide chains with flexible linkages within the backbone act as reversible phase. High molecular weight (*M*_*n*_) is demanded to form physical crosslinks as fixed phase of thermoplastic shape memory polyimide, and the relationship between *M*_*n*_ and glass transition temperature (*T*_*g*_) is explored. Thermoset shape memory polyimide shows higher *T*_g_ and storage modulus, better shape fixity than thermoplastic counterpart due to the low-density covalent crosslinking, and the influence of crosslinking on physical properties are studied. The mechanism of high temperature shape memory effects based on chain flexibility, molecular weight and crosslink density is proposed. Exposure to thermal cycling from +150 °C to −150 °C for 200 h produces negligible effect on the properties of the shape memory polyimide, and the possible mechanism of high and low temperature resistant property is discussed.

**S**hape memory polymers (SMPs) can be deformed into a temporary shape and then return to the original shape under an external stimulus, and the shape memory effects are attributed to the coexistence of one phase allowing for shape fixity and the other phase allowing for reversibility, where the polymer goes from low to high molecular mobility^1^,^2^,^3^,^4^,^5^,^6^,^7^,^8^. Among the various external stimuli such as electrical^9^, light^10^,^11^, and electromagnetic fields^12^, temperature variation has been the main activation stimulus due to the intrinsic thermal phase transition occurred in polymers^13^,^14^,^15^. The majority of the research reports are focused upon SMPs with relatively low to medium shape transition temperature for applications in surgical materials^16^, drug delivery^17^,^18^,^19^, actuators^20^, and so on^21^,^22^.

SMPs with different controllable shape recovery temperatures are desired to fulfill the demands of different environments, and high temperature SMPs have potential applications in various fields requiring high temperature shape memory effects, such as deployable space structures, shape morphing structures, smart jet propulsion system, high temperature actuators, *et al.*^7^,^23^. It is obvious that during most of the operating time, the environmental temperature of high temperature SMP is not mild. A typical example is thermal cycling from +150 °C to −150 °C takes place between the sun-facing and shadow-facing sides of the spacecraft in the orbit. The high and low temperature thermal cycling is the first and foremost crucial problem for space structures, although the severe environment is also characterized by high vacuum, ionizing radiation, UV radiation, atomic oxygen, debris, and so on^24^. Many polymers will suffer from embrittlement at low temperature and degradation at high temperature^25^. If the SMP structures are required to operate for an extended periods of time in harsh environments such as space, the most basic demand is that they can maintain their performances against thermal cycling^24^. Unfortunately, the commercial epoxy and styrene-based SMPs with low to medium glass transition temperature (*T*_*g*_) undergo vast deterioration in shape memory performance after the thermal cycling (unpublished work of our group). Although there have been several reports about high temperature SMPs in recent years^26^,^27^,^28^,^29^,^30^,^31^, there is no report about SMPs that can withstand the high and low temperature thermal cycling.

Aromatic polyimides (PI) have been widely used in aerospace and spacecrafts due to their high *T*_*g*_, high thermal stability, high tensile strength, excellent radiation shielding capability and low creep^32^,^33^,^34^,^35^,^36^,^37^. PI have been used as flexible cables, bushings, bearings, socket or constructive parts besides their common applications as adhesive coating and film-forming materials^38^. Accordingly, shape memory polyimide that combines shape memory effects with the unusual properties of PI are expected to extend applications in various fields. *T*_*g*_ of SMP should be higher than the environmental temperature to avoid premature trigger of shape. Meanwhile, it will be inconvenient to trigger the shape when *T*_*g*_ is too high^26^. Therefore, shape memory polyimides with different *T*_*g*_s are required to satisfy the temperature demands of different environments. Here we report new shape memory polyimide samples with adjustable *T*_*g*_ ranging from 229 to 243 °C, which show excellent thermal cycling resistant properties. The polyimide is synthesized by polycondensation of bis phenol A dianhydride (BPADA) and 4,4′-diaminodiphenyl ether (ODA), and the aromatic polyimide chains possessing thermal stable but flexible linkages within the backbone act as the reversible phase of the shape memory process. Molecular weight has great influence on the physical properties of polymer^39^,^40^,^41^, and high molecular weight is necessary to form physical crosslinks as the permanent phase of thermoplastic shape memory polyimide^27^. The critical number average molecular weight (*M*_*n*_) that determines the shape memory effect is obtained, and the relationships between *M*_*n*_ and *T*_*g*_ are explored. Thermoset SMPs possess chemical resistance, higher storage modulus and higher *T*_*g*_ than the thermoplastic ones due to the covalent crosslinking, and they are preferable for some harsh environments^42^,^43^,^44^. Here thermoset shape memory polyimide samples are fabricated, and the influence of covalent crosslinking on their physical properties are studied. The possible mechanism of high temperature shape memory effects of the polyimide based on chain flexibility, molecular weight and crosslink density is proposed.

The effects of thermal cycling from +150 °C to −150 °C on the thermomechanical properties and shape memory performances of the shape memory polyimide are investigated. The shape memory polyimides possess thermal cycling resistant properties, and the possible reason is discussed.

## Results

### Molecular weight and structure of thermoplastic polyimide

**Figure 1:**
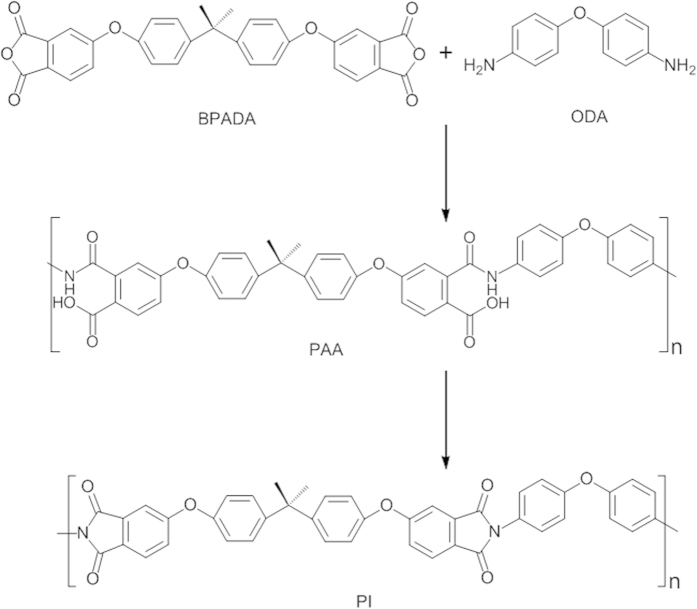
Synthesis of the shape memory polyimide. BPADA and ODA form viscous poly(amic acid) (PAA) at room temperature, and PAA transforms into polyimide by thermal curing. Molecular weight can be varied by changing the molar ratio of BPADA/ODA.

The shape memory polyimide is synthesized by polycondensation of BPADA and ODA, and the schematic illustration for the polycondensation is shown in Fig. 1. The shape memory polyimide samples are semi-transparent, and *M*_*n*_ can be adjusted by changing the molar ratio of BPADA/ODA, and the samples with different *M*_*n*_ are labeled as A0, A1, A2, A3 and A4, respectively, as manifested in Table 1. Fourier transform infrared spectroscopy (FTIR) spectrum of A0 is shown in Figure S1. The peaks at 1780 (C=O, asymmetric stretching) and 1718 cm^−1^ (C=O, symmetric stretching) are the typical frequencies of imides, and the C-N-C absorption at 1373 (stretching vibration) also confirm the formation of imides. There is no evidence of carbonyl absorbances in the range 1795–1820 cm^−1^ and 921–934 cm^−1^, characteristic of isoimides. The carbonyl stretching frequency in an inter-molecular imide linkage near 1670 cm^−1^ is not observed, either^45^. The results demonstrated that the sample is fully imidized within the detection limit of IR.

It is observed that A0 with a stoichiometric ratio possesses the highest *M*_*n*_ of 43.9 kg/mol, and the stoichiometric imbalances lead to decrease in *M*_*n*_, as manifested in Table 1. It is observed that there is a critical *M*_*n*_ of 24.5 kg/mol, and samples with higher molecular weight exhibit excellent shape memory performances. The sample with lower *M*_*n*_ of 22.1 kg/mol (*M*_*w*_ = 37.6 kg/mol) shows a rather low shape fixity of 65% when the applied temperature is *T*_*g*_ + 20 °C, and the shape fixity can reach up to 83% at *T*_*g*_ + 50 °C. But the sample is apt to get breaches that will cause permanent harm at these high temperatures. Therefore, from the viewpoint of shape memory performances, the applied temperature does not have much influence on the critical molecular weight.

An important advantage of SMP is their large strains, and the polyimide with *M*_*n*_ higher than 24.5 kg/mol can produce strain more than 100%. The film of the sample with *M*_*n*_ = 22.1 kg/mol will be broken when the strain is about 30%. Therefore, from the viewpoint of large recoverable strains, the applied strains does not produce obvious effects on the critical molecular weight, either. For samples with much lower molecular weight, such as *M*_*n*_ = 19.2kg/mol (*M*_*w*_ = 31.6 kg/mol), *M*_*n*_ = 16.9 kg/mol (*M*_*w*_ = 28.5 kg/mol) and *M*_*n*_ = 9.8 kg/mol (*M*_*w*_ = 18.4 kg/mol), they cannot form continuous films, but formed fragments.

### Thermomechanical properties of thermoplastic polyimide

**Figure 2:**
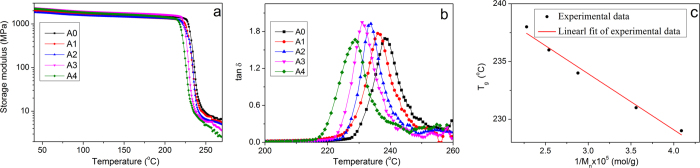
Thermomechanical properties of thermoplastic shape memory polyimides. (**a**) Tensile storage modulus (**b**) loss factor (tan *δ*) versus temperature and (**c**) *T*_*g*_ versus reciprocal *M*_*n*_ of the polyimides.

The thermomechanical properties are very important for the applications of SMP, and those of the thermoplastic shape memory polyimide samples are obtained with dynamic mechanical analysis (DMA), and the tensile storage modulus (*E*′) versus temperature are shown in Fig. 2a. It is observed that *E*′ of the polyimide decreases slowly with the increase of temperatures in the glassy state, and there is not a strong dependence of *E*′ on *M*_*n*_ in glassy state, similar to other thermoplastic polyimide^36^. There is a sharp drops of *E*′ in the vicinity of the softening point, and *E*′ of A0 at 40 °C, 220 °C and 260 °C are 2.1 GPa, 1.4 GPa and 7.1 MPa, respectively. The high *E*′ in glassy state is due to the elastic potential while the low *E*′ in rubbery state is due to the entropy elasticity caused by micro-Brownian movement, and the large difference in *E*′ is necessary for the polymer to exhibit shape memory effects^7^,^28^. There is a plateau in rubbery state, and the shapes of the elastic plateaus of different polyimide samples are similar to each other, indicating that that the physical crosslinks are mainly formed by chain entanglements^7^. The well-defined rubbery plateau is important in the recycle thermal processing of the material, where stable thermal-mechanical properties are required.

DMA is an effective and sensitive method to determine *T*_*g*_ of polyimide and the DSC curve is shown in the supplementary information (Figure S2). The loss factor (tan *δ*) versus temperature of the shape memory polyimides are shown in Fig. 2b. There is an increase of *T*_*g*_ with the increase of *M*_*n*_, as A0 with the highest *M*_*n*_ show *T*_*g*_ of 238 °C and A4 with the lowest *M*_*n*_ show *T*_*g*_ of 229 °C. The higher *T*_*g*_ is mainly associated with a higher degree of chain entanglement, a common phenomenon in polymers^39^,^40^. The relationship between *T*_*g*_ and *M*_*n*_ of the shape memory polyimide is correlated with Equation 1.


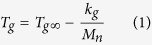


*T*_*g∞*_ is *T*_*g*_ of the sample with infinite molecular weight, and *k*_*g*_ is the parameter explaining the *M*_*n*_ dependence of *T*_*g*_ for the material^46^,^47^. Figure 2c shows the experimental data and their linear fit according to the Fox-Flory equation, and the experimental *T*_*g*_ data are consistent with the predicted ones, exhibiting an adjusted R-square of 0.981. The extrapolation shows that the polyimide with infinite molecular weight would have a delineated *T*_*g*_ of 248.5 °C and *k*_*g*_ of −4.86 × 10^5^ K mol.g^−l^.

### Thermal stability of thermoplastic shape memory polyimides

The thermal stability of the thermoplastic shape memory polyimide are examined with thermal gravimetric analysis (TGA) analysis, and the weight loss versus temperature are shown in Supplementary information (Figure S3). The weight loss of 5% is taken as the effective decomposition temperature (*T*_*d*_), and *T*_*d*_ of the samples are listed in Table 1. It can be seen that *T*_*d*_ of the thermoplastic shape memory polyimide ranges from 486 to 493 °C, and they show an increase with the increase of *M*_*n*_. The pyrolysis left more than 50% carbonaceous char from the aromatic group at 800 °C for each sample, which means that the shape memory polyimide samples are highly thermal stable.

### Shape memory properties of the thermoplastic polyimides

Shape fixity (*R*_*f*_) and shape recovery (*R*_*r*_) are two important factors in determining the shape memory performances of SMP^7^. *R*_*f*_ is the ability of the switching segments to hold the applied mechanical deformation during this process, and it is calculated using Equation 2.


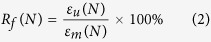


Here *ε*_*u*_ denote the strain in the fixed temporary shape, *ε*_*m*_ denote the strain after the stretching step (before cooling). *R*_*r*_ is the ability of the materials to recover to its original shape, which is calculated using Equation 3.


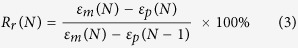


Here *ε*_*m*_*, ε*_*p*_ and N denote the strain after the stretching step (before cooling), the strain after recovery, and the cycle number, respectively.

**Figure 3:**
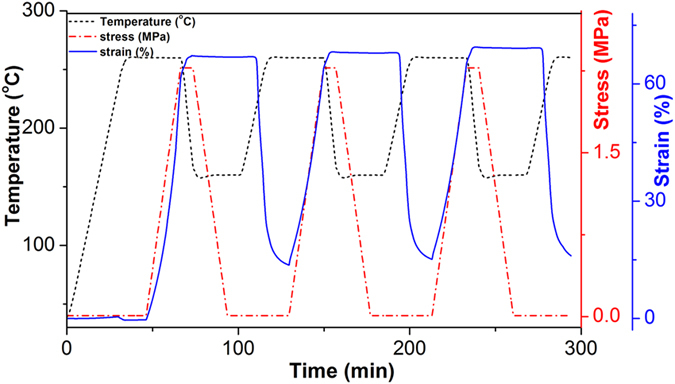
Cyclic shape memory processes of shape memory polyimide. Two-dimensional demonstration of change in strain, stress and temperature with time in stress-controllable consecutive tensile shape memory processes of A0.

*R*_*r*_ and *R*_*f*_ are characterized with consecutive shape memory cycles, and the cycles of A0 are shown in Fig. 3. It is observed that *R*_*f*_ of the three cycles are 99.6%, 99.7% and 99.7%, respectively, which means that the shape is almost completely fixed, and the results are consistent from one cycle to another. *R*_*r*_ is 80.2% for the first cycle, 97.3% and 97.2% for the second and third cycles. The difference in *R*_*r*_ between the first and the following cycles is generally attributed to residual strain resulting from the processing history of the sample and the plastic deformation took place within the first cycle^31^. As the reproducibility of *R*_*f*_ and *R*_*r*_ for each sample is good, the values of the second cycle are employed as the shape memory parameters for the samples, and they are summarized in Table 1. It is observed that all the thermoplastic shape memory polyimide samples show excellent shape memory performances with *R*_*f*_ higher than 97% and *R*_*r*_ higher than 98%, mainly due to the large difference in high modulus storage below *T*_*g*_ and excellent rubber elasticity above *T*_*g*_^7^.

The consecutive shape memory cycles also show excellent shape memory performances whether the strain is increased to 100% or decreased to 15%, as shown in the Supplementary information as Figure S4 and Figure S5, respectively. When the recovery temperature is increased to *T*_*g*_ + 50 °C, the polyimide still exhibit high *R*_*f*_ and *R*_*r*_, as shown in the Supplementary information (Figure S6). But one serious problem is that at this high temperature, the samples are apt to get fractured even they are treated with extreme care and patience. At a higher temperature, the polymer may get deformed seriously and unsuitable for use. Therefore, we do not recommend the process of the shape memory polyimide samples at too high temperatures.

### Swelling of thermoset shape memory polyimide

Thermoset SMPs are expected to find applications in many harsh environments, as they are intrinsically chemical resistant and possess higher tensile strength than the thermoplastic counterparts. In the current research, tris(4-aminophenyl)amine (TAP) with three amine functional groups is employed as crosslink agent to form thermoset shape memory polyimides. It is observed that while the thermoplastic shape memory polyimides can be dissolved in dimethylacetamide (DMAc), the TAP covalent crosslinked shape memory polyimide swelled in DMAc, indicating that crosslinking has taken place. The thermoset shape memory polyimide samples with different TAP content are labeled as B1, B2, B3 and B4, as manifested in Table 2. The gel content and swelling ratio results are shown in Supplementary information (Table S1). The gel content increases with the increase of crosslinker, and reaches 100% for samples with the molar ratio of crosslinker more than 5%. The swelling ratio decreases from 12.68 for sample with 1% crosslinker to 1.56 for samples with 6% crosslinker. The results indicate that more TAP leads to higher chemical crosslink densities, which results in a decrease in swelling by volume percentage^48^.

### Thermomechanical properties of thermoset shape memory polyimides

**Figure 4:**
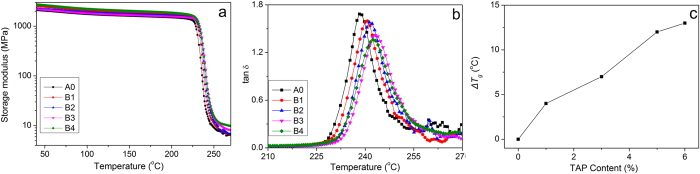
Thermomechanical properties of thermoset shape memory polyimides. (**a**) Tensile storage modulus (**b**) loss factor and (**c**) difference in *T*_*g*_ (*ΔT*_*g*_) between thermoset and corresponding thermoplastic counterpart polyimides. A0 is employed as reference.

The storage modulus of the thermoset shape memory polyimides versus temperature are characterized with DMA, and the results are shown in Fig. 4a with A0 as reference. The *E*′ of every thermoset polyimide at 40 °C is higher than 2.2 GPa, exceeding that of A0, which means that covalent crosslinking can affect their mechanical properties. There is not a linear relationship between the content of TAP and the modulus in glassy state, similar to the aspartimide-based SMPs^26^. *E*′ in the rubbery plateau increases with the increase of crosslink density, as shown in the inlet to Fig. 4a. *E*′ at *T*_*g*_ + 20 °C is 7.6 MPa for A0 without crosslinker and 10.5 MPa for B4 with 6% crosslinker. *E*′ of the thermoset polyimide samples at rubbery state ranges from 7.8 to 10.5 MPa, relatively high for an unreinforced polymer. The increase in *E*′ of thermoset shape memory polyimides indicate that covalent crosslinking can enhance the absolute amount of mechanical energy stored and stress recovered for a unit strain, consistent with the theory of rubber elasticity^28^, and the systematic study on their energy storage properties will be carried out in the future.

The loss factor of the shape memory polyimide are shown in Fig. 4b, and *T*_*g*_ of A0 is lower than those of others, and *T*_*g*_ of the thermoset samples range from 240 to 243 °C. Thermoset polyimides show higher *T*_*g*_ than their thermoplastic counterparts, and the difference between their *T*_*g*_ (*ΔT*_*g*_) as shown in Fig. 4c. It is observed that *ΔT*_*g*_ get higher with the increase of TAP content, from 4 °C for B1 to 13 °C for B4 Fig. 4c. The increase in *T*_*g*_ results from disappearance of chain ends and formation of chemical crosslinks, as there would be greater difficulty for chain slippage with increased binding sites or decreased free volume^7^,^28^. The effective crosslink density that causes the change can be calculated with DiMarzio equation as Equation 4.


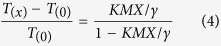


where *T*_*(x)*_ is *T*_*g*_ of the covalent crosslinked polyimide, *T*_*(0)*_ is *T*_*g*_ of the corresponding thermoplastic counterparts, *K* is often taken as 1.3 × 10^−23^, *M* is the molecular weight of the imides repeat unit, *X* is the effective crosslink density, *γ* is the number of flexible bonds per repeat unit^49^. The calculated crosslink density using DiMarzio’s theory increases with the increase of crosslinker content, and ranges from 6.87 × 10^18^ to 2.21 × 10^19^ g^−1^, as summarized in Table 2. The degree of crosslinking in the thermoset polyimides with different TAP contents is 82%, 48%, 48% and 44%, respectively.

### Network structures of the thermoset polyimide

**Figure 5:**
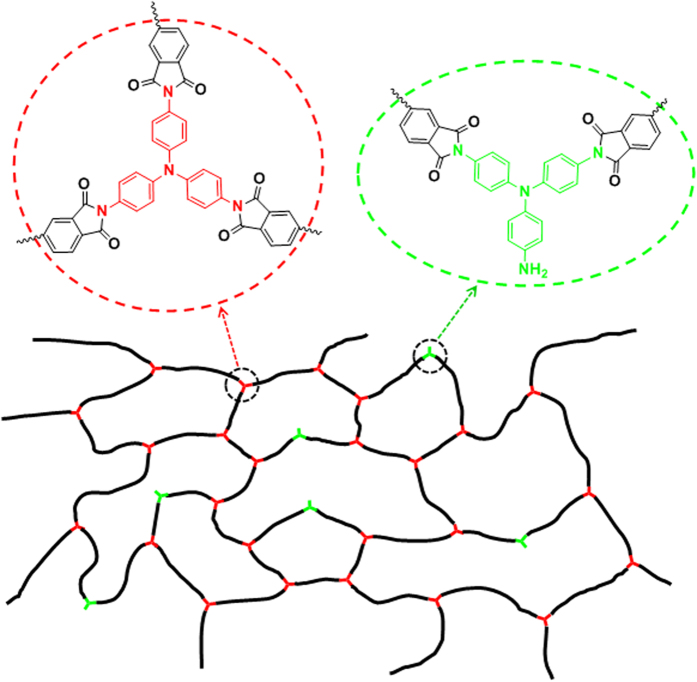
Schematic illustration for the structure of thermoset shape memory polyimide. The black lines represents the polyimide chains, the red Y-type symbols represents TAP acting as crosslinkers and green Y-type symbols represents TAP acting as chain extenders.

It has been reported that for the crosslinker molecules with multiple functional groups, not all but part of the functional group are covalently connected with the polymer chains due to the space hindrance and reaction activity^50^,^51^,^52^. It is obvious that neither effective crosslink density nor *M*_*n*_ of the shape memory polyimide is proportional to TAP content, which suggest that TAP acts as crosslinkers and chain extender to form thermoset shape memory polyimide, consistent with other reports^50^,^51^,^52^. The chemical covalent crosslinks create a network topology on the molecular scale analogous to that created by physical entanglement on the mesoscale^7^, and the schematic illustration for the structure of thermoset shape memory polyimides is manifested in Fig. 5.

### Thermal stability of thermoset shape memory polyimides

The thermal stability of the thermoset shape memory polyimide is studied with TGA, and the weight loss versus temperature is shown in the supplementary information (Figure S7). *T*_*d*_ of the thermoset samples are in the range of 493–501 °C (shown in Table 2), the pyrolysis left about 50% carbonaceous char from the aromatic group at 800 °C for each sample, which means that the thermoset shape memory polymers are highly stable.

### Shape Memory performances of thermoset polyimides

The shape memory performances of the thermoset polyimide are studied with the same procedures of the thermoplastic polyimide, and the shape fixity and shape recovery are summarized in Table 2. The thermoset shape memory polyimdes show shape fixity and shape recovery nearly 100%. The thermoset shape memory polyimides exhibit higher *R*_*r*_ than thermoplastic counterparts, and this is mainly due to the loss in physical crosslink integrity caused by mechanical deformation^7^.

**Figure 6:**
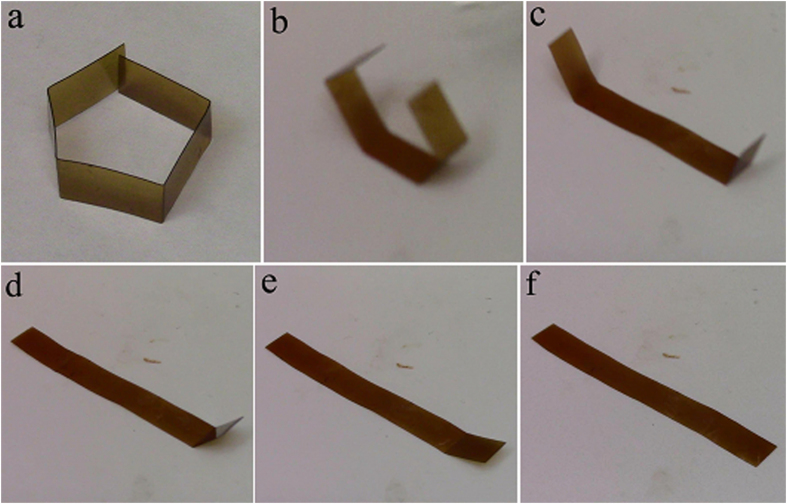
Shape recovery process of the shape memory polyimide. (**a**) shows the deformed shape of the sample B2, and (**b**–**f**) show the shape recovery process on 240 °C hot-stage at 1, 2, 3,4 and 6 s.

The shape memory process of thermoset polyimide sample is also demonstrated more visually by deforming the sheet into a pentagon shape on the surface of hot stage at *T*_*g*_, and the contemporary shape is equilibrated for 5 minutes, and then it is fixed by removing the sample from the hot-stage to ambient. Upon reheating on the same hot-stage, the material returns to the original form. The typical images showing the shape recovery process of B2 are shown in Fig. 6, and the movie showing the shape recovery process is attached as Video 1. The process is repeated fifteen times with no damage observed, and every time the sample appears to recover fully to the initial shape. Other deformation modes like bending also show similar results.

### Influence of high and low temperature on the thermomechanical properties

**Figure 7:**
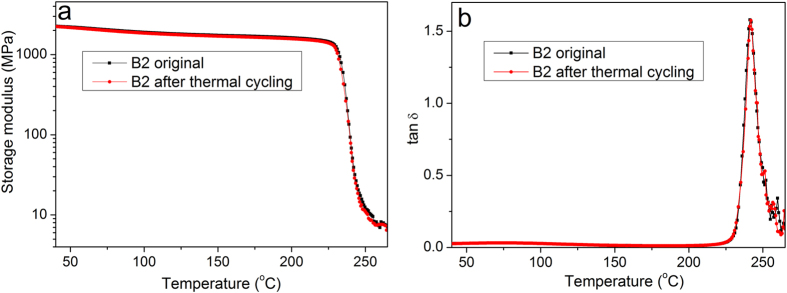
Thermomechanical properties of the shape memory polyimide before and after thermal recycling. (**a**) Tensile storage and (**b**) loss factor (tan *δ*) versus temperature before and after thermal cycling.

The effects of high and low temperature thermal cycling on the physical properties of shape memory polyimide are presented by measurements the thermomechanical properties. The storage modulus and loss factor versus temperature of B2 before and after being exposed to high and low thermal cycling are shown in Fig. 7a,b, respectively. It is observed that *E*′ at *T*_*g*_–20 °C is 1490 and 1456 MPa for the sample with and without thermal cycling, showing a decrease of 2.3%. Meanwhile, *E*′ at *T*_*g*_ + 20 °C is 7.8 and 7.6 MPa, showing an increase of 2.6%, as shown more clearly in the inlet to Fig. 7a. Therefore, the storage modulus of B2 seems to be unaffected by the thermal cycling from +150 °C to −150 °C for 200 h. The loss factor are almost identical to each other without discernible difference in *T*_*g*_. These results demonstrate that the high and low temperature thermal cycling have no obvious effect on the thermomechanical properties of the thermoset shape memory polyimides.

### Influence of high and low temperature on shape memory properties

**Figure 8:**
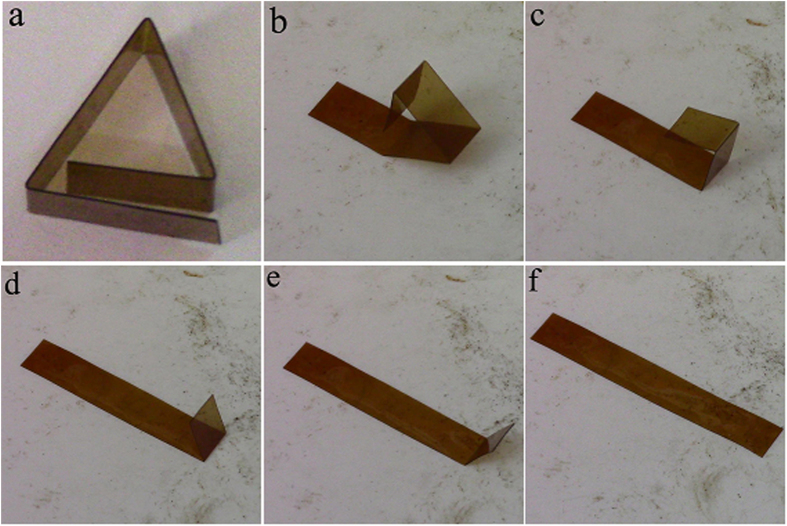
Shape recovery process of shape memory polyimide after thermal annealing. (**a**) shows the deformed shape and (**b**–**f**) show the shape recovery process of B2 after thermal cycling on 240 °C hot-stage at 1, 2, 3, 4 and 5 s.

For the shape memory polyimide sample B2 before and after thermal cycling, the shape fixity is 99.8% and 99.5%, while the shape recovery is 99.7% and 99.6%, respectively. The typical shape recovery process of the sample after thermal cycling is more visually shown in Fig. 8, and the movie is in attached as Video 2. Almost fully shape recovery and shape fixity are possible, which means that the high and low temperature thermal cycling on these conditions do not lead to deterioration in the material’s ability to lock in or recover strain.

Radiations cause challenge to the space structures, and the γ-ray radiation resistant properties of the shape memory polyimides were studied with the radiation dose of 1 × 10^6^ Gy, equal to the accumulated dose of two years that a spacecraft will experience. The morphology, color, weight, and shape memory behavior of the shape memory polyimides have no discernable difference after exposure to γ-ray radiations, and the shape memory process of B2 after exposure is attached as Video 3.

## Discussion

The shape memory effect of SMP is generally explained by dual-state mechanism, in which the molecular chains are regarded as the reversible phase, and the nodes of macromolecule segments attributed to physical or chemical crosslinks are regarded as the permanent phase^1^,^5^,^43^ SMPs with *T*_*g*_ lower than 110 °C are mainly composed of polymers with flexible main chains, while SMPs with high *T*_*g*_ are all characteristic of the rigid chains comprising phenyl rings and flexible linkages^26^,^27^,^28^,^29^,^30^,^31^. The shape memory polyimide here is obtained by polycondensation of BPADA/ODA, where the isopropylidene and ether linkage within the backbone provide some flexibility to the highly aromatic polyimide structures, and the molecular chains act as reversible phase. For SMP polyimide with stoichiometric ratio of dianhydride/diamine, the sample based on BPADA/ODA shows *T*_*g*_ of 238 °C, while the samples based on 4,4′-oxydiphthalic dianhydride (ODPA)/ODA, 4,4′-(hexafluoroisopropylidene) diphthalic anhydride (6FDA)/1,3-bis(3-aminophenoxy)benzene (BAB) and BPADA/2,2-bis[4-4(-aminophenoxy) phenyl] propane (BAPP) exhibited *T*_*g*_ of 260.7 °C, 222 °C and 212 °C, respectively^27^,^28^,^53^. Molecular structures of the shape memory polyimide samples with typical *T*_*g*_s are demonstrated in the Supplementary information (Figure S8). For dianhydrides, ODPA possess one ether linkage, BPADA possesses one isopropylidene and two ether linkages, while 6FDA possesses one hexafluoroisopropylidene in the backbone. For diamines, BAPP possesses one isopropylidene and two ether linkages, BAB possesses two ether linkages, and ODA possesses one ether linkage in the backbone. There is an obvious increase in flexibility of the rigid polyimide chains following the sequence of ODPA/ODA>BPADA/ODA>6FDA/BAB>BPADA/BAPP. The relative rigidity of the polymer backbone can affect dynamics through the glass transition^42^, and the different polyimides exhibit *T*_*g*_ in the sequence of chain rigidity.

For thermoplastic shape memory polyimide, high *M*_*n*_ with a critical value of 24.5 kg/mol is needed to form physical crosslink of thermoplastic shape memory polyimide. Low *M*_*n*_ produces short chains that can’t form chain entanglements, while high *M*_*n*_ produces long chains to produce chain entanglements that act as the main factor to form physical crosslinks of shape memory process, and aryl interactions also contribute partially to the physical crosslinks. The thermoset shape memory polyimide shows higher *T*_*g*_ and better shape memory performances than the thermoplastic counterpart due to the covalent crosslinking, and the effective crosslink density varies from 6.87 × 10^18^ to 2.21 × 10^19^ g^−1^. The mechanism of high temperature shape memory effects of the polyimide based on chain flexibility, molecular weight and crosslink density is proposed, which may stimulate further research on new high temperature SMPs.

The highly aromatic rigid chains are reported to be responsible for the high and low temperature resistant ability of polyimide like the commonly used Kapton^32^. Although the main chains of the shape memory polyimide in this research is more flexible than that of Kapton, it is still composed of aromatic rigid chains, and the relatively rigid chain mainly account for the +150 °C to −150 °C thermal cycling resistant properties. These shape memory polyimides can also withstand the irradiation of γ-ray under 1 × 10^6^ Gy. This research has offered a likely candidate resin for deployable and morphing structures in harsh environments such as low earth orbit (LEO), which represents an important step in the applications of SMP structures for extreme conditions like space.

## Methods

### Materials

BPADA (99%) and ODA (98%) are purchased from Sigma-Aldrich Co., TAP (98%) is purchased from TCI chemicals, and the chemicals are used as received. Dimethylacetamide (DMAc) is purchased from Kemiou Chemical Reagent Co. Ltd. and distilled with activated 4 Å molecular sieves under reduced pressure.

### Synthesis of thermoplastic polyimide

All the lab instruments and glasswares for the synthesis are strictly dry, and the synthesis were carried out in a constant-temperature room, where the temperature is kept at 20 °C by air conditioners. ODA and 20 ml DMAc are added to a three-necked flask and then stirred under dry nitrogen for 25 mins. 4 mmol BPADA is fed into the flask in batches within 1 hr, and intense mechanical stirring is executed at room temperature for 22 hr to form highly viscous PAA. The flask is transferred into a vacuum drying chamber and heated under vacuum at 50 °C for 2.5 hr to remove the bubbles. The PAA is poured on the glass plate and ungoes a step-wise imidization curing process at 70 °C/2 hr, 100 °C/2 hr, 150 °C/2 hr, 190 °C/2 hr, 230 °C/2 hr and 280 °C/2 hr. The polyimide samples are detached away from glass in deionized water, and was heated at 180 °C for 3 hr to remove the water. Polyimide with different *M*_*n*_ values were prepared by varying the molar ratio of BPADA to ODA, and the formula of the samples are shown in Table 1.

### Synthesis of covalent crosslink polyimide

The highly viscous PAAs are obtained with the same procedures, and TAP was then added to the PAA solution, where the total amount of amine groups (2 in one ODA molecule and 3 in one TAP molecule) is equal to that of anhydride group (2 in one BPADA molecule).The mixture is stirred for another 5 hr, and then undergoes the vacuum evaporation and thermal imidization as mentioned above.

### Molecular weight and structure characterization

The molecular weight of the polyimide samples were determined by size exculsion chromotography (SEC) using Waters 2414 equipped with a refractive index detector in a mixture of N,N-dimethylformamide (DMF) and 0.05 M lithium bromide at 36 °C. The structure of the shape memory polyimide is studied by Fourier transform infrared spectroscopy (FTIR) on Thermo Nicolet Nexus 870.

### Thermomechanical characterization

The storage modulus and loss factor (tan *δ*) of the shape memory polyimide specimens versus temperature are characterized by dynamic mechanical analysis (DMA) on Netzsch Q800. The samples are cut into rectangular slabs with dimensions of 36 mm × 3 mm × 0.15 mm, and DMA tests were conducted in tensile mode at the frequency of 1 Hz with a heating rate of 3 °C/min.

### Thermal stability measurement

Differential scanning calorimetry (DSC, NETZSCH STA 449C) measurements were used to characterize the change in the thermal properties of the polymers at a heating rate of 10 °C/min. The thermal stability of the polyimide films was performed by thermal gravimetric analysis (TGA) under N_2_ environment on a *Mettler*-*Toledo TGA/DSC instrument* at a heating rate of 10 °C/min.

### Shape memory characterization

A flat sheet of 0.15 mm thick was employed to show the shape memory process of the shape memory polyimide more visually. The film was fixed into a pentagon on the surface of hot stage, and after each step of deformation an equilibration of is performed, and the shape was fixed by removal from the hot stage after equilibration. The shape recovery on the surface of hot stage was recorded by a digital video (Canon VIXIA HF R500).

### Strain fixing and recovery rates measurement

Shape memory cycles to assess strain fixing and recovery rates are measured using the controlled force mode on TA INSTUMENT Q800. The procedure for the cyclic tensile tests includes the following steps: (a) heating a sample to *T*_*g*_ + 30 °C at 10 °C/min, (b) applying a force that would elongate the sample; (c) reducing the temperature to *T*_*g*_ − 70 °C at 10 °C/min, (d) force removal, (e) heating at 10 °C/min to *T*_*g*_ + 30 °C. Once recovery of the sample reached a constant value the cycle was repeated by applying the same force used in step (b).

### Thermal cycling process

The maximum and the minimum operating temperatures for the thermal cycling are +150 °C and −150 °C, respectively. One cycle takes about 90 minutes, and the whole experiment last for about 200 h.

**γ-Ray** Radiation Resistant Characterization. The γ-ray radiation resistant capability of the shape memory polyimide sample was studied with ^60^Co-γ as the radiation source at the rate of 300 Gy.min^−1^ at room temperature, and the total dose is 1 × 10^6^ Gy.

## Additional Information

**How to cite this article**: Xiao, X. *et al.* Shape memory polymers with high and low temperature resistant properties. *Sci. Rep.* **5**, 14137; doi: 10.1038/srep14137 (2015).

## Supplementary Material

Supplementary Information

Video 1

Video 2

Video 3

## Figures and Tables

**Table 1:** Physical properties of thermoplastic shape memory polyimide.

ODA/BPADA	Title	*M*_*n*_(kg/mol)	*M*_*w*_(kg/mol)	*T*_*g*_ (°C)	*E*′ (MPa) at *T*_*g*_ − 20 °C	*E*′ (MPa) at *T*_*g*_ + 20 °C	*T*_*d*_ (°C)	*R*_*f*_ (%)	*R*_*r*_ (%)
1	A0	43.9	92.2	238	1407	7.6	493	99.7	97.3
0.9850	A1	39.4	75.3	236	1292	7.3	492	99.7	97.6
0.955	A2	34.7	64.5	234	1308	6.1	490	99.2	97.0
0.925	A3	28.1	51.8	231	1607	5.8	488	98.5	97.2
0.910	A4	24.5	41.2	229	1403	4.8	486	98.0	97.0

Samples with lower *M*_*n*_, no shape memory properties.

**Table 2:** Physical properties of thermoset shape memory polyimide.

TAP (%)	Title	*T*_*g*_(°C)	*ΔT* (°C)	DiMarzio-X (g^−1^)	*E*′ (MPa) at*T*_*g*_ − 20 °C	*E*′ (MPa) at*T*_*g*_ + 20 °C	*T*_*d*_(°C)	*R*_*f*_(%)	*R*_*r*_ (%)
0	A0	238	0	0	1407	7.6	493	99.7	97.3
1	B1	240	4	6.87 × 10^18^	1580	7.8	495	99.9	98.2
3	B2	241	7	1.19 × 10^19^	1492	7.9	497	99.8	99.5
5	B2	243	12	2.03 × 10^19^	1465	8.8	501	99.9	99.8
6	B4	242	13	2.21 × 10^19^	1673	10.5	499	99.9	99.7
