# Supplementary material for: Shape memory polymers with high and low temperature resistant properties
Source: Sci Rep. 2015 Sep 18;5:14137. doi: 10.1038/srep14137 (PMC4585657; doi:10.1038/srep14137)
Supplement: Supplementary Information [file srep14137-s1.pdf]

Supplementary Information

Shape memory polymers with high and low temperature resistant properties

Xinli Xiao[1,2], Deyan Kong[2], Xueying Qiu[2], Wenbo Zhang[2],Yanju Liu[3], Shen Zhang[2], Fenghua Zhang[1], Yang Hu[2], and Jinsong Leng[1*]

1.Centre for Composite Materials and Structures, Harbin Institute of Technology (HIT), No. 2 YiKuang Street, PO Box 3011, Harbin 150080, People's Republic of China. 2.Department of Chemistry, Harbin Institute of Technology (HIT), P.O. Box 301, No. 92 West Dazhi Street, Harbin 150001, People's Republic of China. 3.Department of Astronautical Science and Mechanics, Harbin Institute of Technology (HIT), P.O. Box 301, No. 92 West Dazhi Street, Harbin 150001, People's Republic of China. Correspondence and requests for materials should be addressed to J.S.L.(email: lengjs@hit.edu.cn).

Figure Captions

Supplementary Figure S1. FTIR of the shape memory polyimide.

Supplementary Figure S2. DSC spectra of thermoplastic shape memory polyimides.

Supplementary Figure S3. TGA spectra of thermoplastic shape memory polyimides.

Supplementary Table S1. Gel content and swelling ratio of thermoset shape memory polyimides.

Supplementary Figure S4. Cyclic shape memory processes of shape memory polyimide with different strains.

Supplementary Figure S5. Cyclic shape memory processes of shape memory polyimide with small strains.

Supplementary Figure S6. Cyclic shape memory processes of shape memory polyimide at high temperatures.

Supplementary Figure S7. TGA spectra of thermoset shape memory polyimide with thermoplastic A0 as reference.

Supplementary Figure S8. Molecular structures of the typical shape memory polyimide samples with different glass transition temperatures.

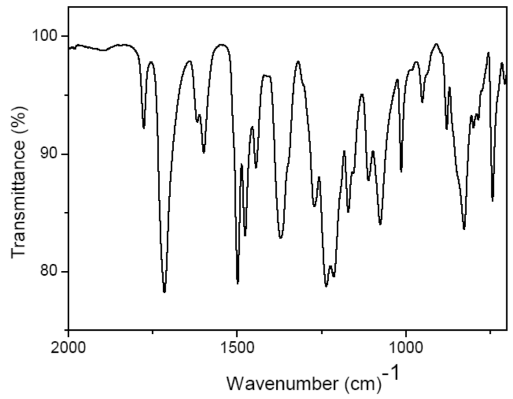

Figure S1. FTIR of the shape memory polyimide. The IR spectra of typical thermoplastic shape memory polyimide A0.

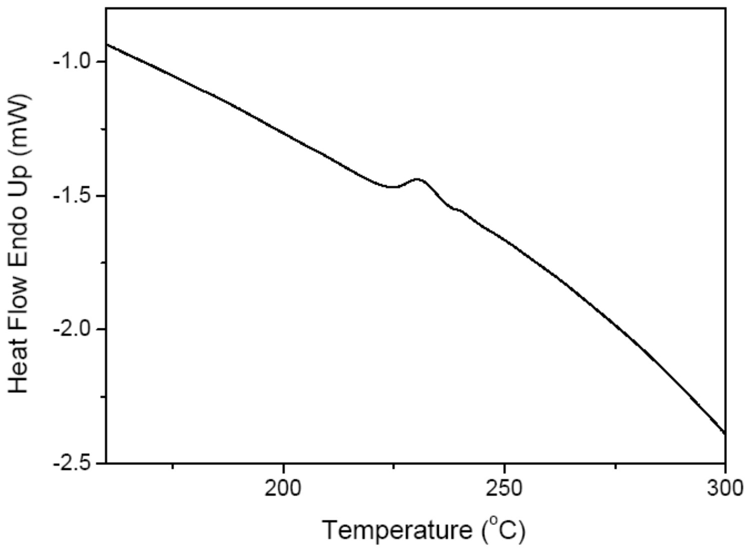

Figure S2. DSC of the shape memory polyimide. The DSC spectra of A0 is measured in N_2 atmosphere at 10 ºC/min.

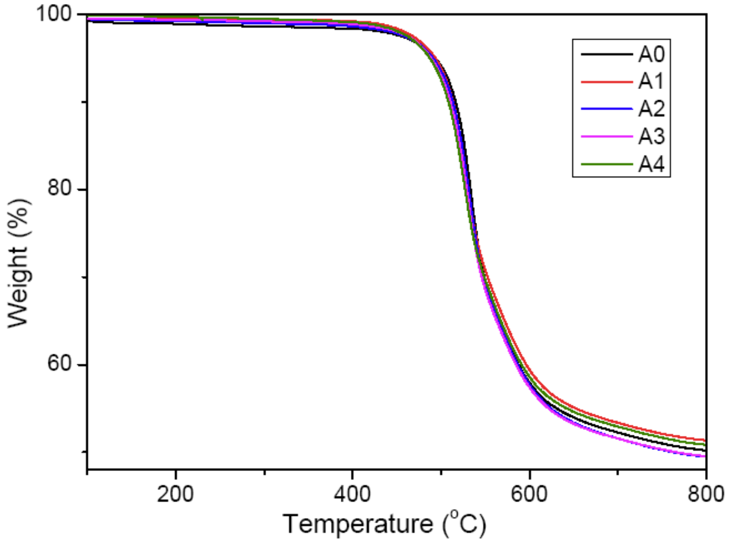

Figure S3. TGA spectra of thermoplastic shape memory polyimides. The loss of weight versus temperature of the thermoplastic shape memory polyimide under N_2 atmosphere at a heating speed of 10 ºC/min.

Swelling tests were performed to gain a basic understanding of the amount of crosslinking that had occurred during the polymerization process. These samples were weighed for their initial mass (m_i) and separated into vials where they were soaked in DMAc. The samples were repeatedly weighed out of the solvent until their masses stabilized to a saturated swollen weight (m_s). These samples were then dried in a desiccator under vacuum for 5 minutes and then transferred to an oven at 120 ℃ overnight, and the next day the dry masses (m_d) were determined. Equation (1) and (2) to determine gelation and swelling degree, respectively.

The gel content (G) of the films was calculated from Equation S1:

$$G = \frac{m_d}{m_i} \quad \text{(S1)}$$

The swelling degree (Q) of the films in NMP was calculated by Equation S2:

$$Q = \frac{crosslinked\ polyimide + absorbed\ DMAc}{crosslinked\ polyimide} \quad \text{(S2)}$$

There is a continuous increase of the gel content with increase of TAP concentration up to about 5 %, after which the content levels off.

TAP (%)	Gel Content (%)	Swelling ratio
0	0	-
1	23.1	12.68
3	78.7	5.62
5	100	2.78
6	100	1.56

Table S1. Gel content and swelling ratio of thermoset shape memory polyimide.

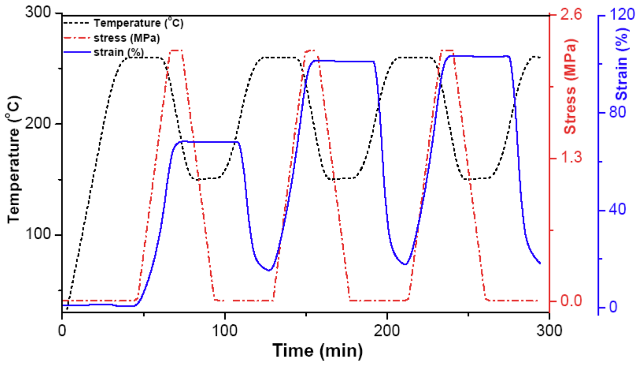

Figure S4. Cyclic shape memory processes of shape memory polyimide with different strains. The first cycle strain is 68 %, the second cycle is 105 %, and the third cycle is 108 %, and the change in strain, stress and temperature with time in stress-controllable shape memory processes of A0 are demonstrated.

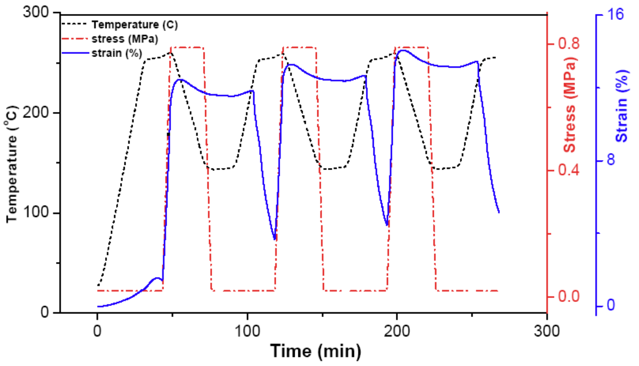

Figure S5. Cyclic shape memory processes of shape memory polyimide with small strains. The strain is in the range of 13 %- 15 %, and the change in strain, stress and temperature with time in stress-controllable shape memory processes of A0 are demonstrated.

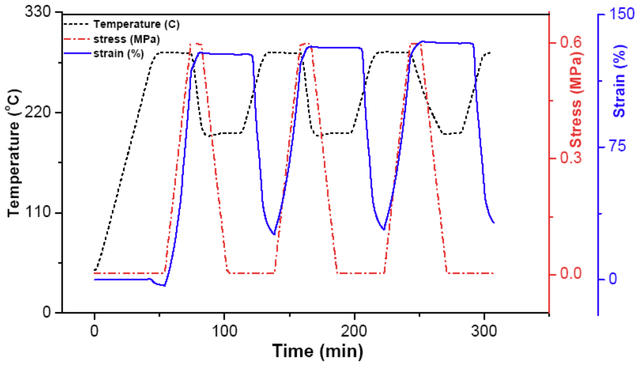

Figure S6. Cyclic shape memory processes of shape memory polyimide at high temperatures. The shape recovery temperature is 290 ºC, and the change in strain, stress and temperature with time in stress-controllable shape memory processes of A0 are demonstrated.

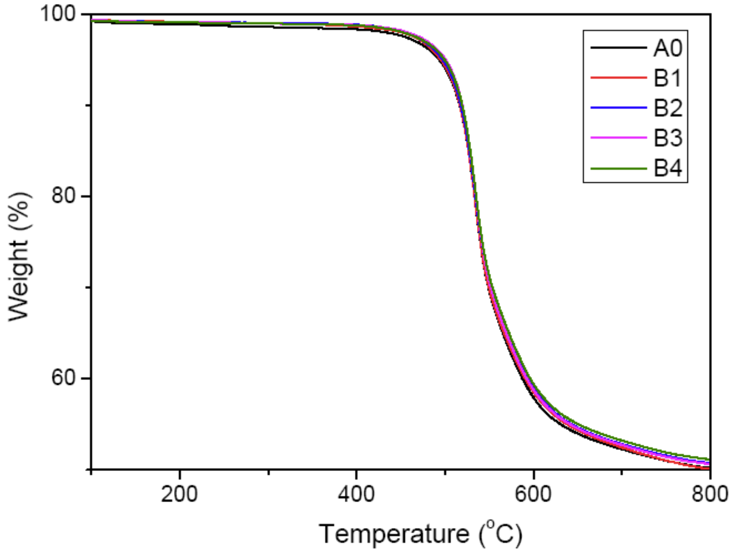

Figure S7. TGA spectra of thermoset shape memory polyimide. The loss of weight versus temperature of the thermoset shape memory polyimide under N_2 atmosphere at a heating speed of 10 ºC/min.

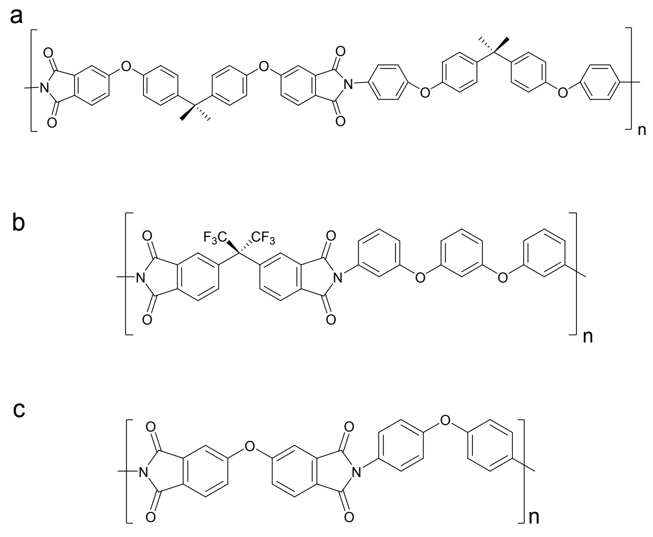

Figure S8. Molecular structures of the typical shape memory polyimide samples with different glass transition temperatures. (a) Polyimide based on BPADA/BAPP, [1] (b) polyimide based on 6FDA/BAB, [2] and (c) polyimide based on ODPA/ODA. [3]

REFERENCES:

(1) Yoonessi, M.; Shi, Y.; Scheiman, D. A.; Lebron-Colon, M.; Tigelaar, D. M.; Weiss, R. A.; Meador. M. A. Graphene Polyimide Nanocomposites; Thermal, Mechanical, and High-Temperature Shape Memory Effects. *Acs Nano* **2012,** *6,* 7644–7655.

(2) Koerner, H.; Strong, R. J.; Smith, M. L.; Wang, D. H.; Tan, L. S.; Lee, K. M.; White, T. J.; Vaia. R. A. Polymer Design for High Temperature Shape Memory: Low Crosslink Density Polyimides. *Polymer* **2013,** *54,* 391–402.

(3) Wang, Q. H.; Bai,Y. K.; Chen, Y.; Ju, J. P.; Zheng, F.; Wang, T. M. High Performance Shape Memory Polyimides Based on π-π Interactions. *J. Mater. Chem. A,* **2015,** 3, 352-359.
